# let-7e replacement yields potent anti-arrhythmic efficacy *via* targeting beta 1-adrenergic receptor in rat heart

**DOI:** 10.1111/jcmm.12288

**Published:** 2014-04-24

**Authors:** Xin Li, Baoqiu Wang, Hairong Cui, Yue Du, Yang Song, Lei Yang, Qi Zhang, Fei Sun, Dan Luo, Chaoqian Xu, Wenfeng Chu, Yanjie Lu, Baofeng Yang

**Affiliations:** a Department of Pharmacology (Key Laboratory of Cardiovascular Medicine Research, Ministry of Education; State-Province Key Laboratories of Biomedicine-Pharmaceutics of China), Harbin Medical UniversityHarbin, Heilongjiang, China; b Department of Surgery, First Affiliated Hospital of Harbin Medical UniversityHarbin, Heilongjiang, China

**Keywords:** acute myocardial infarction, let-7e, β_1_-AR, anti-arrhythmia

## Abstract

Beta-adrenoceptor (β-AR) exerts critical regulation of cardiac function. MicroRNAs (miRNAs) are potentially involved in a variety of biological and pathological processes. This study aimed to investigate the role of miRNA let-7e in the up-regulation of β_1_-AR and arrhythmogenesis in acute myocardial infarction (AMI) in rats. β_1_-AR expression was significantly up-regulated and let-7a, c, d, e and i were markedly down-regulated in the infarcted heart after 6 and 24 hrs myocardial infarction. Forced expression of let-7e suppressed β_1_-AR expression at the protein level, without affecting β_1_-AR mRNA level, in neonatal rat ventricular cells (NRVCs). Silencing of let-7e by let-7e antisense inhibitor (AMO-let-7e) enhanced β_1_-AR expression at the protein level in NRVCs. Administration of the lentivirus vector containing precursor let-7e (len-pre-let-7e) significantly inhibited β_1_-AR expression in rats, whereas len-AMO-let-7e up-regulated β_1_-AR relative to the baseline control level, presumably as a result of depression of tonic inhibition of β_1_-AR by endogenous let-7e. Len-negative control (len-NC) did not produce significant influence on β_1_-AR expression. Len-pre-let-7e also profoundly reduced the up-regulation of β_1_-AR induced by AMI and this effect was abolished by len-AMO-let-7e. Importantly, len-pre-let-7e application significantly reduced arrhythmia incidence after AMI in rats and its anti-arrhythmic effect was cancelled by len-AMO-let-7e. Notably, anti-arrhythmic efficacy of len-pre-let-7e was similar to propranolol, a non-selective β-AR blocker and metoprolol, a selective β_1_-AR blocker. Down-regulation of let-7e contributes to the adverse increase in β_1_-AR expression in AMI and let-7e supplement may be a new therapeutic approach for preventing adverse β_1_-AR up-regulation and treating AMI-induced arrhythmia.

## Introduction

Acute myocardial infarction (AMI) or heart attack as a result of blockage of a coronary artery in the clinical setting is a common cause of mortality and morbidity worldwide. It occurs after a prolonged period of myocardial ischemia, which can result in cardiac electrophysiological disturbance, haemodynamic disorder, metabolic disorders, necrosis and apoptosis of cardiomyocytes [1,2], accompanied by abnormal alterations of gene expression such as aberrant regulation of a number of ion channels [3] and β-adrenoceptors (β-AR) [4,5], and reduction in connexin43 [6,7], which can often lead to arrhythmias and acute and chronic heart failure.

β-adrenoceptor plays a pivotal role in regulating cardiac function and heart rate (HR). And β-AR blocker is commonly used to treat patients with arrhythmia and heart failure [8,9]. To date, three subtypes of β-AR have been identified pharmacologically in the heart: β_1_-AR, β_2_-AR and β_3_-AR. β_1_-AR and β_2_-AR are the major subtypes that modulate cardiac contractility and HR by stimulating the G-protein/adenylate cyclase/protein kinase A pathway [10,11]; however, β_3_-AR produces negative inotropic effects by coupling to inhibitory G proteins [12]. In physiological conditions, β_1_-AR and β_2_-AR subtypes are expressed at a ratio of 70:30 [13,14]. However, β-AR expression can be affected by many pathological processes of the heart such as AMI and chronic heart failure. For example, β-AR expression is increased in the acute phase of myocardial infarction [4,5]; in contrast, it is decreased in chronic heart failure [15].

Dynamic expression regulation of β-AR and functional desensitization are considered adaptive or protective mechanisms in the heart. Regulation of β-AR expression involves a variety of factors and pathways in cardiac cells. Ihl-Vahl *et al*. reported that subtype-selective increase in β_1_-AR induced by AMI is associated with a transcriptional up-regulation, and occupation or activation of β_1_-AR is not involved in this subtype-specific up-regulation of β_1_-AR [4]. Bengtsson *et al*. reported that a protein synthesis inhibitor cycloheximide regulates β_1_-AR gene expression by acting on repressive elements in the promoter region of the gene in brown adipocytes [16]. Calcium mobilization and protein kinase C activation participate in the down-regulation of β-AR in activated B cells by allogenic stimulation and/or soluble factors [17].

MicroRNAs (miRNAs) are a family of single-stranded non-coding RNAs that post-transcriptionally regulate the translation of protein-coding genes by targeting to the 3′ untranslated regions (3′UTRs) of mRNAs. Recently, accumulating evidence has demonstrated that miRNAs play a critical role in pathophysiology of cardiovascular diseases, such as hypertrophy, heart failure and arrhythmias, *etc*. [18]. Inhibition of miR-208a prevented pathological myosin switching and cardiac remodelling [19]. Overexpression of miR-133 or miR-101 attenuated cardiac hypertrophy [20] or cardiac fibrosis [21] respectively. MiR-1 overexpression contributed to slow conduction, membrane depolarization [22], atrio ventricular block [23] and afterdepolarizations [24]; while miR-1 inhibition was involved in atrial fibrillation (AF) [25]. Studies also showed that miR-21 [26], miR-26 [27], miR-328 [28], miR-133 and miR-590 [29] participated in the process of AF by controlling the expression of their specific gene targets.

In this study, we, for the first time, displayed the involvement of let-7, a conserved and abundant miRNA in the heart [30], in the up-regulation of β_1_-AR in AMI in rats, which provides new insight into the mechanisms for regulation of β_1_-AR expression and overexpression of miRNA let-7e potentially inhibited AMI-induced arrhythmia in rat. This indicates that targeting miRNA let-7e may be a promising therapeutic strategy for modulating β_1_-AR.

## Materials and methods

### Animals

Healthy male Wistar rats (200 ± 20 g; Vitalriver, Beijing, China) used in this study were kept under standard animal room conditions (temperature 21 ± 1°C; humidity 55–60%) with food and water *ad libitum* for 1 week before experimental interventions. All experimental procedures were in accordance with, and approved by the Institutional Animal Care and Use Committee of the Harbin Medical University.

### Rat model of AMI

Rats were anaesthetized with ketamine (60 mg/kg) and xylazine (6 mg/kg). Tracheal cannula was performed with a polyethylene tube and ventilated with the TOPO small animal Ventilato (Kent, OH, USA), and then the chest was opened through the fourth intercostal space and propped ribs by a rib spreader. The pericardium was opened carefully to expose the heart. The left anterior descending coronary artery (LAD) was ligated using a 5/0 silk thread to create infarction of the LV free wall. Cardiac infarction was confirmed by apparent S-T segment elevation in ECG and cyanosis of the myocardium. The sham procedure consisted of a superficial suture in the epicardium of the LV.

### MiRNA microarray and data analysis

We performed miRNA expression profiling with the heart samples from with or without AMI (6 hrs) rats. RNA samples 5 μg were labelled with the Exiqon miRCURY Hy3/Hy5 power labelling kit and hybridized on the miRCURY LNA Array (version 11.0) station. Scanning was performed with the Axon GenePix 4000B microarray scanner. GenePix pro version 6.0 was used to read image and analyse raw intensity. The threshold value for significance used to define up-regulation or down-regulation of miRNAs was a fold change >1.5 or <0.5.

### Western blot

Total protein was extracted with RIPA Lysis Buffer (Beyotime, Shanghai, China) mixed with 1% proteinase inhibitors, and degenerated by admixing with 5× loading buffer (Beyotime) at 100°C for 5 min. Extracted protein samples (120 μg from NRVCs and 60 μg from tissues) were separated in 10% SDS-PAGE and blotted to nitrocellulose membrane. The blots were blocked with 5% non-fat milk overnight at 4°C, probed with a primary antibody to β_1_-AR (1:20 dilution; Santa Cruz Biotechnology Inc., Santa Cruz, CA, USA), or to β_2_-AR (1:1000 dilution; Abcam, Cambridge, MA, UK), or to GAPDH (1:1000 dilution; Jinshan, Shanghai, China), or to β-actin (1:500 dilution; Jinshan) in 5% non-fat milk, and incubated at 4°C overnight. The membranes were washed with PBS-T and PBS, and then incubated with secondary antibody (LI-COR Bioscience, Lincoln, NE, USA) for 1 hr at room temperature. Finally, western blot bands were collected by Imaging System (LI-COR Biosciences) and quantified with odyssey v1.2 software by measuring intensity in each group with GAPDH as an internal control, but for the ischaemia tissues using β-actin as an internal control [31]. The results were expressed as fold changes by normalizing the data to the values from the control group.

### Quantitative reverse transcription-PCR (qRT-PCR)

After experimental treatment, total RNA samples were isolated from cultured NRVCs and cardiac tissues using Trizol reagent (Invitrogen, Carlsbad, CA, USA) according to manufacturer's protocol. RNA (0.5 μg) was then reverse transcribed using High-Capacity cDNA Reverse Transcription Kit (Applied Biosystems, Foster City, CA, USA) to obtain first-strand cDNA. Levels of let-7a/c/d/e/i, miR-1 and β_1_-AR mRNA were determined using SYBR Green I incorporation methods on ABI 7500 fast Real Time PCR system (Applied Biosystems), with U6 as an internal control of miRNA or GAPDH as an internal control of β_1_-AR mRNA. The sequences of primers used in the qRT-PCR experiments (Invitrogen, Shanghai, China) were listed in Table S1.

### Construction of plasmid carrying the 3′UTR of β_1_-adrenergic receptor (ADRB1) and luciferase assay

Targetscan predicts the presence of a putative binding site for let-7 in the 3′UTR of ADRB1 mRNA, the gene encoding β_1_-AR, which is highly conserved among mammals. A segment containing the let-7 miRNA binding sites flanked by the Hand lll and Sac I restriction sites and a scramble sequence as a negative control (NC) were synthesized by Invitrogen. The sequences were inserted separately into the pMD18T-simple vector (Invitrogen), and then transferred into the pMIR-REPORT™ Luciferase miRNA Expression Reporter Vector (Ambion, Austin, TX, USA). pRL Renilla Luciferase Reporter vector (pRL-TK, Promega, Madison, WI, USA) was used as an internal control. The plasmids and miRNAs were transfected grouply with X-tremeGENE siRNA transfection reagent (Roche, Mannheim, Germany) into HEK293 cells. The dual luciferase reporter assay kit (Promega) and the GlouMax biological fluorescent tester were used to detect the activity of the luciferase 36 hrs after transfection. The results were expressed as fold changes by normalizing the scaled data. The sequence of human and rat ADRB1 3′UTR inserted into the vectors were as follows: The human β_1_-AR 3′UTR (position 704-711 of human ADRB1 3′UTR, 160 base pairs): 5′-CGAGCTCTTAAGCTCTTCTTGGAACAAGCCCCACCTTGCTTTCCTTGTGTAGGGCAAACCCGCTGTCCCCCGCGCGCCTGGGTGGTCAGGCTGAGGGATTTCTACCTCACCTGTGCATTTGCACAGCAGATAGAAAGACTTGTTTATATTAAGCTTGGG-3′. The rat β_1_-AR 3′UTR (position 712-719 of rat ADRB1 3′UTR, 139 base pairs): 5′-CGAGCTCCTCGGTGGTCCTGCTGTGGGTCCTCTACCTCACTCTGTGCATATTGCACAGCAAGATAGAAAGACTTGTTTATATTAAACAGCTTATTTATGTATCAATATTAGTTGGAAGGACCAGGCGCTGAAGCTTGGG-3′.

### Cardiomyocytes isolation and culture from neonatal rats

The neonatal Wistar rats of 1–3 days old were disinfected by 75% ethanol. The chest was opened to expose the heart. The heart was isolated and sheared into 1- to 3-mm pieces. The cardiac tissues were digested into single cardiomyocytes by 0.25% trypsin. Dispersed cells were placed in high glucose DMEM containing 10% fetal bovine serum (FBS) and 1% benzylpenicillin-streptomycin to terminate the digestion procedure. Pooled cell suspensions were centrifuged and resuspended in DMEM supplemented with 10% fetal bovine serum, and 100 U/ml penicillin and 100 μg/ml streptomycin. The resuspension was transferred onto a 100-mm culture dish for 30 min., then changed to a new dish, and repeated for three times. This procedure allowed for preferential attachment of fibroblasts to the bottom of the culture dish and left non-adherent cardiomyocytes in cell suspension. The cells were plated into a 6-well plate at a density of 1 × 10^6^ per well, and incubated at 37°C in a humidified atmosphere of 5% CO_2_ and 95% air.

### Synthesis of let-7c/d/e mimics and anti-let-7c/d/e antisense inhibitors

Rno-let-7c/d/e and their antisense oligonucleotides (AMO-let-7c/d/e) were synthesized by RiboBio (RiboBio, Guangzhou, China). In addition, a scrambled RNA was used as a NC (sequence: 5′-UUCUCCGAACGUGUCACGU-3′) that designed according to the Blast search of the human/rat/mouse genomic by RiboBio. let-7c: 5′-UGAGGUAGUAGGUUGUAUGGUU-3′; AMO-let-7c: 5′-AACCAUACAACCUACUACCUCA-3′; let-7d: 5′-AGAGGUAGUAGGUUGCAUAGUU-3′; AMO-let-7d: 5′-AACUAUGCAACCUACUACCUCU-3′; let-7e: UGAGGUAGGAGGUUGUAUAGUU; AMO-let-7e: 5′-AACUAUACAACCUCCUACCUCA-3′. All pyrimidine nucleotides in the NC or miRNA mimics were substituted by their 2′-O-methyl analogues to improve RNA stability. Transfection of synthesized RNAs was accomplished by using X-tremeGENE siRNA Transfection Reagent (Roche). Thirty-six hours after transfection of let-7e mimic (100 nM) or AMO-let-7e (200 nM), the NRVCs were used for qRT-PCR and Western blot analysis.

### Construction of HIV-1-based lentivirus carrying pre-let-7e

Lentivirus vectors expressing mature let-7e, anti-miRNA-oligo of let-7e (AMO-let-7e) or NC sequence were constructed by Invitrogen (Invitrogen). Briefly, the precursor sequence of let-7e and its antisense fragment were synthesized by Invitrogen. The synthesized fragments were annealed and inserted into the pcDNA™ 6.2-GW/EmGFP-miR vector (Invitrogen). Then, lentivirus plasmid was constructed by BP and LR recombination into pLenti6.3/TO/V5-DEST vector (Invitrogen) through the Gateway recombination technology. The constructed pLenti6.3/TO/V5-DEST plasmid and an optimized mix of the three packaging plasmid (pLP1, pLP2 and pLP/VSVG; Invitrogen, China) were cotransfected into 293FT procedure cell line by liposome reagent to package lentivirus. The virus liquid was collected and concentrated 48 hrs after cotransfection, and the titre of the lentivirus liquid was determined.

### *In vivo* lentivirus infection and beta-blocker administration

Thoracotomy was performed in the fourth left intercostal space to expose the heart of rats. Virus-containing solution (20 μl, 1 × 10^8^ TU) including len-NC, precursor let-7e (len-pre-let-7e), AMO-let-7e (len-AMO-let-7e) or len-pre-let-7e and len-AMO-let-7e was injected using an insulin syringe into LV wall of rat heart. Rats in the sham and MI groups underwent the same procedures but received the same volume of saline (20 μl) with the constructs. Rats in β-blocker groups received non-specific β-AR blocker propranolol (10 mg/kg/day, Sigma-Aldrich, St. Louis, MO, USA) and specific β_1_-AR blocker metoprolol (80 mg/kg/day; Sigma-Aldrich) by intragastric administration for 7 days. Ligation of LAD was performed at day 7 after injection and administration. All surgery equipments were sterilized to ensure the minimum level of infection. Rats were given penicillin (1 × 10^5^ Units/day, im) for 7 days. After surgery, the rats were given food and water *ad libitum*.

### Ultrasound imaging and haemodynamics parameter measurements

Rats were anaesthetized and pre-thoracic fur was removed by a Nair™ depila-tory cream (Church & Dwight Co., Inc., Princeton, NJ, USA). Haemodynamic measurements were performed by the Vevo®2100 High-Resolution Imaging system (Visual Sonics, Toronto, ON, Canada) in rats 6 hrs after ligation or 7 days after treatments with len-pre-let-7e or len-AMO-let-7e. Rats were positioned on a RatPad (part of the VisualSonics Vevo Integrated Rail System II) equipped with integrated heater. Body temperature was maintained at 37°C. Pre-warmed Aquasonic Clear® Ultrasound Gel (Parker Laboratories, Inc., Fairfield, NJ, USA) was used as a coupling agent between the ultrasound scan-head and the skin. Two-dimensional targeted M-mode traces were obtained with the transducer (MicroScan MS 250-0206) held immobilized. LV end-diastolic and end-systolic wall thickness and LV internal diameter were measured from at least three consecutive cardiac cycles. Ejection fraction (EF) and fractional shortening (FS) were calculated based on the Vevo®2100 High-Resolution Imaging system (Visual Somics).

### Electrocardiogram (ECG) monitoring and arrhythmia scoring

As described above, rats were infected with len-NC, precursor let-7e (len-pre-let-7e), AMO-let-7e (len-AMO-let-7e) or len-pre-let-7e + len-AMO-let-7e, or administrated with either β-AR blocker propranolol or metoprolol. Ligation of LAD was performed on day 7 after infection and administration. Then, standard lead II digital ECG tracings were recorded for evaluation of arrhythmias using the BL-420F bio-function experiments system (Taimeng, Chengdu, China). Arrhythmia score was evaluated from an ECG recorded during 30 min. period after the LAD ligation using Score F as described by Curtis and Walker [32,33], based on the analysis of premature ventricular contractions (PVCs), ventricular tachycardia (VT), ventricular fibrillation (VF), ventricular fibrillation terminated spontaneously (SVF), and VF termination was not spontaneous (NVF). PVCs were identified by the presence of a premature QRS complex; VT was classified as three or more consecutive PVCs. The arrhythmia scoring system F is as follows: 0 = <50 PVCs, 1 = ≥50 PVCs, 2 = 1–5 episodes of VT, 3 = ≥6 episodes of VT, 4 = 1 SVF or 1 episode of NVF or both, 5 = 2–5 episode of NVF, 6 = ≥5 episode of NVF.

### Data analysis

All data were statistically analysed by using one-way anova followed by Turkey's multiple comparison test. Data of arrhythmia score were statistically analysed by using Wilcoxon signed-rank test. Differences were considered as statistically significant when *P* < 0.05. Data are presented as mean ± SD or mean ± SEM.

## Results

### Up-regulation of β_1_-AR and down-regulation of let-7 in infarcted hearts

**Fig. 1:**
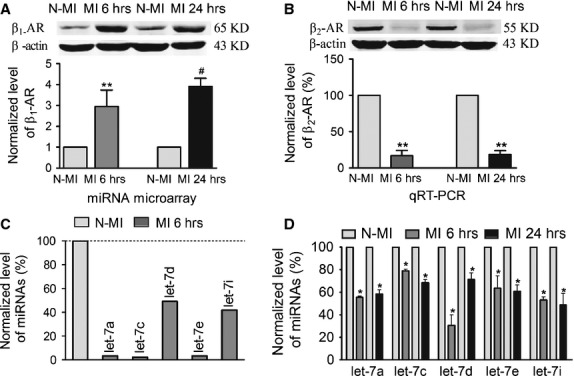
Up-regulation of β_1_-AR and down-regulation of let-7 in rat models of acute myocardial infarction (AMI). (**A**) β_1_-AR protein level in 6 hrs (MI 6 hrs) and 24 hrs (MI 24 hrs) myocardial ischaemia rats, *n* = 5 batches for each group. (**B**) β_2_-AR protein level in 6 hrs (MI 6 hrs) and 24 hrs (MI 24 hrs) myocardial ischaemia rats, *n* = 3 batches for each group. (**C**) microRNA microarray analysis of let-7a, c, d, e and i. (**D**) Real-time PCR analysis of let-7a, c, d, e and i levels. *n* = 3–5 rats for each group, data are expressed as mean ± SD. *P < 0.05 *versus* Ctl; **P < 0.01 MI 6 hrs *versus* Ctl, ^#^*P* < 0.01 MI 24 hrs *versus* Ctl (non-ischaemia area of LV from the same heart).

We first compared the expression levels of β_1_-AR between the infarcted and non-infarcted LV tissues in a rat model of AMI. β_1_-AR expression was up-regulated by 3.0 ± 0.8-fold (*P* < 0.01) at the protein level 6 hrs after AMI (Fig. 1A), compared to non-ischaemic area. Similar results were observed in the infarcted tissue 24 hrs after AMI (Fig. 1A). In contrast to the up-regulation of β_1_-AR, β_2_-AR expression was markedly down-regulated at the protein level 6 hrs (*P* < 0.01) and 24 hrs (*P* < 0.01) after AMI (Fig. 1B), compared to non-ischemic area. These data are consistent with previous reports on selective up-regulation of β_1_-AR in acute ischemic heart [2,3].

We then studied the expression of let-7, a cardiac-enriched miRNA, in the infarcted rat heart with miRCURY Array microarray version 11.0 containing 349 mature rat miRNAs. The let-7 family includes let-7a, b, c, d, e, f, g and i and they all share an identical seed motif thereby presumably possessing the same cellular functions. We found that the levels of let-7a, c, d, e, and i decreased by >50% in the ischaemic tissue 6 hrs after MI, compared with non-ischaemic area (Fig. 1C). Above data were confirmed by qRT-PCR. let-7a expression decreased by 44.6%±1.3%, let-7c by 21.1%±1.5%, let-7d by 69.3%±9.3%, let-7e by 36.2%±10.9% and let-7i by 46.9%±2.9% in the infarcted area of rat heart with 6hrs of AMI (Fig. 1D). Similarly, let-7a expression decreased by 41.6%±3.8%, let-7c by 31.6%±2.9%, let-7d by 28.6%±5.7%, let-7e by 39.2%±5.5% and let-7i by 51.3%±10.3% after 24 hrs of AMI (Fig. 1D).

### Regulation of β_1_-AR by let-7 in cardiomyocytes *in vitro*

**Fig. 2:**
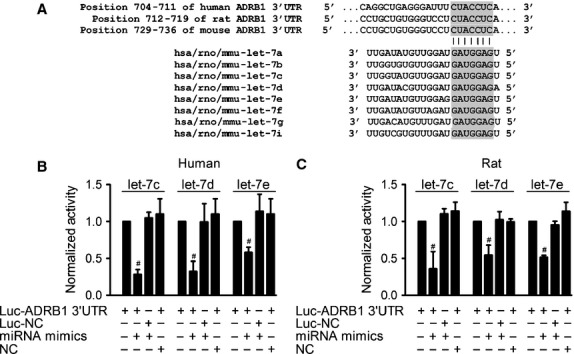
Predicted seed-binding sites of let-7 in β_1_-AR 3′UTR and verification of β_1_-AR as a target for let-7. (**A**) Alignment of the sequences of let-7 family (bottom) with their target sites in the 3′UTRs of human, rat and mouse β_1_-AR mRNA (top). The complementarity is highlighted in grey. Activities of pMIR-REPORT™ luciferase vector carrying luciferase gene and fragment of ADRB1 3′UTR from human (**B**) and rat (**C**) containing the binding sites of let-7c, d and e. The intensity of luciferase was detected 36 hrs after transfection. Data are expressed as mean ± SD, *n* = 3–4 batches of cell; ^#^*P* < 0.01 *versus* Ctl.

The reciprocal alterations of β_1_-AR and let-7 in terms of their expression in AMI suggest a targeting relationship between them. To exploit this notion, we performed miRNA gene target prediction using TargetScan 6.0 database, and we indeed identified a binding site in the 3′UTR of β_1_-AR mRNA for all members of the let-7 family, which is highly conserved among human, rat and mouse (Fig. 2A).

We then experimentally verified the regulation of human and rat β_1_-AR by let-7c, d and e with luciferase activity assay in HEK-293 cells (Fig. 2B and C). Results showed that let-7c, d, and e significantly inhibited luciferase activity elicited by the pMIR-REPORT™ luciferase vector containing their target sequence, but cotransfection with let-7c, d, e and mutant Luc-ADRB1 3′UTR were unable to inhibit the luciferase activity and mutant let-7c, d or e alone did not influence the luciferase activity. All these data indicate the specificity of let-7 action on ADRB1 3′UTR (Fig. 2B and C).

**Fig. 3:**
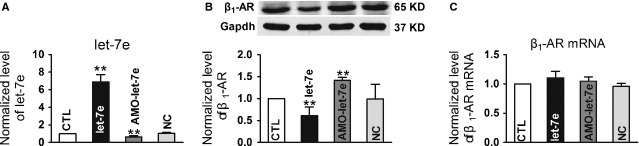
Verification of gain- and loss-of-function of let-7e in cultured neonatal rat ventricular cells (NRVCs). (**A**) Let-7e levels in NRVCs transfected with the let-7e, AMO-let-7e and negative control (NC). (**B** and **C**) β_1_-AR mRNA (*n* = 3 batches of cell) and protein levels (*n* = 4–6 batches of cell) in let-7e, AMO-let-7e and negative control transfected NRVCs. Data are expressed as mean ± SD; **P < 0.01 *versus* Ctl (no treatment).

Efficient transfection of let-7e was verified by 6.9 ± 0.9-fold elevation of this miRNA in NRVCs. On the other hand, AMO-let-7e, a specific inhibitor for let-7e, reduced the let-7e level to below the baseline control level, indicating a knockdown of both exogenous and endogenous let-7e. The NC had no effect on let-7e expression (Fig. 3A).

Overexpression of let-7e significantly inhibited β_1_-AR expression. AMO-let-7e resulted in a higher β_1_-AR protein level than the control group, indicating a relief of tonic inhibition of β_1_-AR by endogenous let-7e in NRVCs. Negative control of let-7e did not show any effect on β_1_-AR expression (Fig. 3B). The expression of β_1_-AR at mRNA level was unaffected by let-7e and AMO-let-7e (Fig. 3C).

### Regulation of β_1_-AR by let-7e *in vivo*

**Fig. 4:**
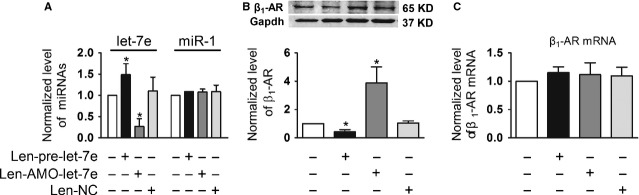
Regulation of β_1_-AR by let-7e *in vivo*. (**A**) Levels of mature miRNA let-7e and miR-1 in the rat heart infected with len-pre-let-7e, len-AMO-let-7e and len-negative control (len-NC). (**B** and **C**) Expression of β_1_-AR at mRNA and protein levels in the rat heart, which were treated with len-pre-let-7e, len-AMO-let-7e or len-NC. Data are expressed as mean ± SD, *n* = 3–4 in each group, *P < 0.05 *versus* sham.

let-7e was increased by 1.5 ± 0.3-fold in the rat hearts administered with len-pre-let-7e compared with the control animals. And as expected, it was decreased in the len-AMO-let-7e group. Len-NC had no effect on let-7e expression (Fig. 4A). We also measured miR-1 expression in the tissue with the same treatments and found no difference in miR-1 expression among the groups, indicating that the observed changes of let-7e expression were specifically elicited by the lentivirus vector carrying the pre-let-7e or AMO-let-7e (Fig. 4A).

Furthermore, we evaluated the effects of let-7e on β_1_-AR expression in the heart. As shown in Figure 4B, β_1_-AR expression was significantly down-regulated in the injected area of the heart in the len-pre-let-7e group, compared with control group. While the len-AMO-let-7e application caused conspicuous increase in β_1_-AR beyond the control level, presumably as a result of inhibition of the receptor by endogenous let-7e. Len-NC did not exert any effects on β_1_-AR expression and len-pre-let-7e had no effect on β_1_-AR mRNA level (Fig. 4C). These results established let-7e as a regulator of β_1_-AR expression in rat heart.

### let-7e inhibits up-regulation of β_1_-AR in ischaemic heart

**Fig. 5:**
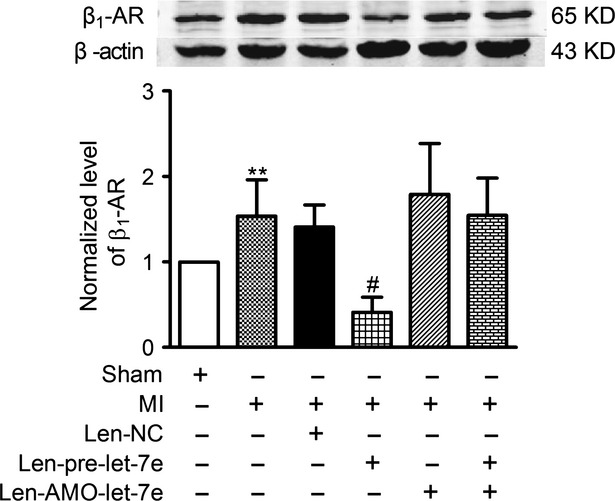
Effects of let-7e on expression of β_1_-AR in ischaemic myocardium in a rat model of AMI. The protein level of β_1_-AR in the ischaemia tissue (6 hrs of ischaemia) pre-treated with len-let-7e, len-AMO-let-7e, len-NC and saline for sham group for 7 days. Date are expressed as mean ± SD, *n* = 5–10 in each group; **P < 0.01 *versus* sham, ^#^*P* < 0.01 *versus* MI.

To investigate the regulation of β_1_-AR by let-7e in the infarcted rats, lentivirus vectors containing pre-let-7e, AMO-let-7e or a scrambled sequence were injected into the LV wall at five points within the expected infarct area 7 days prior to AMI. Of note, len-let-7e profoundly mitigated AMI (6 hrs)-induced β_1_-AR overexpression, and which was abolished by co-treated with len-AMO-let-7e (Fig. 5). Len-AMO-let-7e alone did not significantly affect the ischemic β_1_-AR overexpression. β_1_-AR expression was not changed in the len-NC group (Fig. 5). Indicating that miRNA let-7e decreased the up-regulation of β_1_-AR in AMI rats.

### Anti-arrhythmic effects of let-7e in AMI rats

**Fig. 6:**
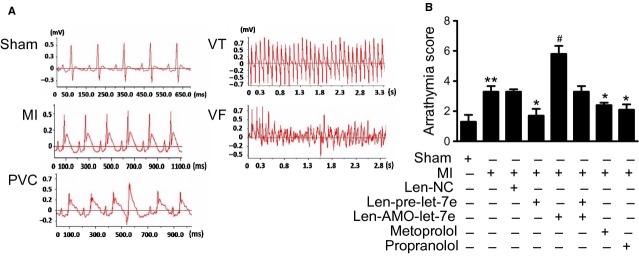
Inhibition of AMI-induced arrhythmia by Let-7e. (**A**) Representative ECG showing sinus rhythm, myocardial ischaemia (MI), premature ventricular contraction (PVC), ventricular tachycardia (VT) and fibrillation ventricular (VF). (**B**) Arrhythmia score in different groups. Data are expressed as mean ± SEM, *n* = 10 rats in each group; ^#^*P* < 0.05 and *P < 0.05 *versus* MI, **P < 0.05 *versus* sham.

Acute myocardial infarction was characterized by induction of cardiac arrhythmias. Electrocardiography recording was performed after ligation of left coronary artery of rat subjected to the pre-treatments with len-pre-let-7e, len-AMO-let-7e or β_1_-AR blocker 7 days ago. Arrhythmia score was calculated from 30 min. period of ECG recording after LAD ligation based on the occurrence of PVCs and episodes of VT and NVF. Notably, len-pre-let-7e infection showed a significant reduction in arrhythmia score from 3.3 ± 0.4 for AMI to 1.7 ± 0.4 (*P* < 0.05). By comparison, rats with treatment of propranolol (10 mg/kg/day), a non-selective β-AR blocker had an arrhythmia score of 2.1 ± 0.3 (*P* < 0.05), and those treated with metoprolol (80 mg/kg/day), a selective β_1_-AR blocker had a score of 2.4 ± 0.2 (*P* < 0.05). All three groups showed significant anti-arrhythmic effects in AMI rats. In contrast, len-AMO-let-7e increased arrhythmia score from 3.3 ± 0.4 to 5.8 ± 0.5 (*P* < 0.05). Len-NC had no effect on the score (Fig. 6B). These data indicate that let-7e has high antiarrhythmic efficacy, which is similar to classic β-blockers. Consistent with arrhythmia score results, len-pre-let-7e significantly reduced occurrence of PVCs from 30.7 ± 3.5 to 16.2 ± 5.7 (*P* < 0.05; Fig. S1A), and decreased episodes of VT from 21.8 ± 7.2 to 3.0 ± 1.4 (*P* < 0.05; Fig. S1B), and reduced episodes of NVF from 0.4 ± 0.2 to 0.0 ± 0.0 (Fig. S1C) in the AMI rats.

### Effects of let-7e on cardiac function and heat rate

Echocardiogram was performed on rats 7 days after treatments with len-pre-let-7e, len-AMO-let-7e or len-NC. Results showed that there were no significant changes in the cardiac function and HR in normal rats among groups (Table 1). These data indicate that local application of let-7e appears not sufficient to make significant effects on the cardiac function and HR.

**Table 1:** Let-7e had no effect on cardiac function and HR in normal rats

Group	Ctl (*n* = 8)	Len-pre-let-7e (*n* = 8)	Len-AMO-let-7e (*n* = 8)	Len-pre-let-7e + Len-AMO-let-7e (*n* = 8)	Len-NC (*n* = 8)
HR (BPM)	345 ± 19	335 ± 45	360 ± 45	349 ± 21	324 ± 67
EF (%)	78.9 ± 3.0	74.4 ± 8.5	73.2 ± 9.1	74.7 ± 10.9	78.5 ± 5.4
FS (%)	48.7 ± 2.9	44.8 ± 7.0	44.0 ± 8.6	45.9 ± 10.2	48.6 ± 5.5

HR, heart rate; EF, ejection fraction; FS, shortening fraction; Len-pre-let-7e, lentivirus vector carrying the precursor let-7e; Len-AMO-let-7e, lentivirus vector was enabled to express anti-miRNA-oligo of let-7e (AMO-let-7e); Len-NC was enabled to express the scramble of let-7e. Data are expressed as mean ± SD.

To valuate cardiac function in AMI rats with different treatments, echocardiogram was performed 6 hrs after LAD ligation. Results showed that len-pre-let-7e, metoprolol and propranolol significantly caused deterioration of cardiac function, reflected by decreased EF and FS in AMI rats (Table 2). Len-AMO-let-7e enhanced EF from 67.9 ± 1.4 to 81.4 ± 2.3 (*P* < 0.05) and FS from 46.8 ± 5.5 to 56.0 ± 5.2 (*P* < 0.05). Both metoprolol and propranolol significantly lowered HR from 355 ± 16 to 280 ± 24 (*P* < 0.05) and from 355 ± 16 to 264 ± 27 (*P* < 0.05) in AMI rats respectively. However, len-pre-let-7e had no significant effect on HR of AMI rats (Table 2).

**Table 2:** Effects of let-7e on HR and cardiac function in acute myocardial infarcted rats

Group	Sham (*n* = 10)	MI (*n* = 9)	len-NC + MI (*n* = 8)	len-pre-let-7e + MI (*n* = 8)	len-AMO-let-7e + MI (*n* = 6)	len-pre-let-7e + len-AMO-let-7e + MI (*n* = 9)	Metoprolol + MI (*n* = 9)	Propranolol + MI (*n* = 10)
HR (BPM)	328 ± 27	355 ± 16	351 ± 16	350 ± 10	355 ± 17	348 ± 21	280 ± 24†	264 ± 27†
EF (%)	77.3 ± 7.8	67.9 ± 1.4*	68.0 ± 13.5	50.7 ± 9.8†	81.4 ± 2.3†	66.9 ± 3.2	49.8 ± 13.6†	36.2 ± 16.8†
FS (%)	58.3 ± 5.6	46.8 ± 5.5*	44.5 ± 6.4	28.1 ± 6.6†	56.0 ± 5.2†	44.4 ± 2.8	29.1 ± 8.8†	24.1 ± 12.3†

HR, heart rate; EF, ejection fraction; FS, shortening fraction; Len-pre-let-7e, lentivirus vector carrying the precursor let-7e; Len-AMO-let-7e, lentivirus vector was enabled to express anti-miRNA-oligo of let-7e (AMO-let-7e); Len-NC was enabled to express the scramble of let-7e. Data are expressed as mean ± SD;

* *P* < 0.05 *versus* Sham;

† *P* < 0.05 *versus* MI.

## Discussion

The aim of this study was to investigate the miRNA mechanisms for the abnormal up-regulation of β_1_-AR and its effect on arrhythmogenesis in the setting of AMI. There are several new findings in this study. First, our data revealed that let-7 was significantly down-regulated, along with selectively increase in β_1_-AR expression, in the infarcted area of LV tissue. Second, we experimentally established β_1_-AR as a target gene for the members of the let-7 family. Third, we demonstrated that let-7e replacement could ameliorate the abnormal up-regulation of β_1_-AR expression in AMI. And finally, let-7e application markedly inhibited arrhythmia incidence in AMI rats. Taken together, it is plausible that deregulation or, specifically, down-regulation of let-7e contributes to the adverse increase in β_1_-AR expression and let-7e supplement shows a potential anti-arrhythmic effect in ischemic heart, which may be a new therapeutic approach for preventing adverse β_1_-AR up-regulation and treating ischemic arrhythmia.

β_1_-adrenoceptor, a predominant subtype of β-AR, exerts a positive inotropic and chronotropic effects in the heart. In certain pathophysiological conditions, β-AR expression is subtype-selective in the heart, such as that β_2_-AR is up-regulated in the transplanted human heart [34], β_1_-AR up-regulated in AMI [4,5] and β_1_-AR down-regulated in chronic heart failure [15]. Acute myocardial infarction is a type of acute coronary syndrome and can lead to decreased cardiac output as a result of impaired cardiac pump function. To compensate for the decreased cardiac output, one of reactions in the infarcted heart is up-regulation of β_1_-AR expression [4,5], but not other type of β-AR. In this study, term β_1_-AR up-regulation refers to increase in the total number of the receptor not including receptor internalization or externalization. Several factors or pathways have been reported to be involved in the regulation of β_1_-AR expression in the heart. IhI-VahI *et al*. reported that AMI induces increase in β_1_-AR subtype as a result of a transcriptional regulation [4], possibly by cAMP pathway [35,36]. Above studies show that the increased β_1_-AR by cardiac ischemia is regulated by the transcription of ADRB1 gene (encoding β_1_-AR) possibly *via* cAMP/PKA/CREB pathway [4,36]. However, other mechanisms for regulation of β_1_-AR expression cannot be ruled out, such as post-transcription regulation of the receptor.

A variety of studies have demonstrated that let-7, an abundant and conserved miRNA, participates in various pathophysiological processes, such as cancer growth and formation [37] and axon regeneration [38]. *In silico* prediction with Targetscan and miRanda showed that ADRB1 is a target of let-7; the seed sequence is conserved among species, such as rat, mouse and human beings. We also demonstrated that β_1_-AR is a target gene for let-7 revealed by luciferase reporter assay and Western blot analysis. We further verified that let-7a, c, d, e, and i were down-regulated in the acute ischemic tissue and forced overexpression of let-7e inhibited β_1_-AR expression and knockdown of this miRNA by AMO-let-7e increased β_1_-AR expression in neonatal rat cardiomyocytes. However, let-7e did not inhibit the mRNA level of ADRB1 gene, indicating that let-7e regulates β_1_-AR expression by disrupting mRNA translation, not by degrading the mRNA. Importantly *in vivo* study, we demonstrated that β_1_-AR expression was regulated by let-7e *via* locally applying this miRNA to rat heart. Notably, len-AMO-let-7e caused an overshoot of β_1_-AR expression relative to the baseline control level, strongly suggesting that this cardiac-enriched miRNA exerts important tonic inhibition of β_1_-AR in the heart.

let-7 family shares the same seed sequence (5′ GAGGUAG 3′) and are highly conserved across species in both their sequences and functions [39]. Study also demonstrated that the let-7 family shares the same downstream targets in human embryonic stem cells [40]. In this study, we freely chose let-7c, d and e from five decreased members of let-7 family for luciferase activity assay. As expected, all three miRNAs significantly inhibited luciferase activity, indicating that ADRB1 3′UTR is direct target of let-7 family. The results from let-7e are most likely applicable to other members of the let-7 family based on their same mechanism of action conferred by their same seed site.

The most important finding is that len-pre-let-7e significantly lowered the incidence of arrhythmia induced by AMI in rats; as expected, len-AMO-let-7e increased the incidence of AMI-induced arrhythmia. Our results also showed that efficiency of its antiarrhythmic effects was very similar with that of classic β-AR blocker propranolol, a non-selective β-blocker, and metoprolol, a selective β_1_-AR blocker, and that are commonly used to treat a variety of cardiac arrhythmia, including ischemic arrhythmia. Cardiac function and HR were not influenced by either len-pre-let-7e or len-AMO-let-7e in normal rats. However, in AMI rats, len-pre-let-7e reduced cardiac function but not affected HR. However, both propranolol and metoprolol inhibited cardiac function and slowed HR. These may suggest that local intramuscular injection of let-7e to LV wall is unable to affect β_1_-AR level in the sinoatrial node that controls HR. The present data indicate that like β-AR blocking agent, let-7e has pronounced antiarrhythmic effect in the setting of AMI.

Previous study demonstrated that only β_1_-AR expression is up-regulated in AMI. However, the mechanisms involved in subtype-selective regulation of β_1_-AR are not fully understood. Several studies showed that adenylyl cyclase and cAMP participate in up-regulation of β_1_-AR in AMI by enhancing transcription of the receptors [35,36,41,42]. Recently, Wang *et al*. [43] reported that let-7f regulates the expression of β_2_-AR, a predominant subtype [44], in lung epithelial H292 cells. On the basis of these data, one would expect that down-regulation of let-7 should also influence expression of β_2_-AR in addition to up-regulation of β_1_-AR. However, it is known that AMI only causes β_1_-AR up-regulation, without altering β_2_-AR. Clearly, mechanisms other than let-7 may also participate in the regulation of β_2_-AR expression in the heart. This study does not provide explanation of this issue. More importantly, our study also showed that let-7e presented a potential antiarrhythmic efficacy in AMI rats as β-AR blocker did. let-7e exerted its beneficial effect mainly through inhibiting the up-regulated β_1_-AR induced by ischemia. However, we definitely could not rule out other molecular targets of let-7, which are involved in its antiarrhythmic effect in the rats with AMI. Our study reveals a new mechanism of β_1_-AR regulated by let-7e and find that let-7e exerts a potential antiarrhythmic effect by targeting β_1_-AR in AMI rats. let-7e might be a promising target for intervention of β_1_-AR in the pathological condition, and let-7e supplement may be a new therapeutic approach for preventing and treating ischaemia-induced arrhythmia.

## Supporting information

**Table S1** Primers used in qRT-PCR experiments.

**Figure S1** Alleviative effect of let-7e on the occurrence of PVCs, VT and NVF induced by acute myocardial infarction.
