# Supplementary figures and images for: let-7e replacement yields potent anti-arrhythmic efficacy via targeting beta 1-adrenergic receptor in rat heart
Source: J Cell Mol Med. 2014 Apr 24;18(7):1334–43. doi: 10.1111/jcmm.12288 (PMC4124018; doi:10.1111/jcmm.12288)

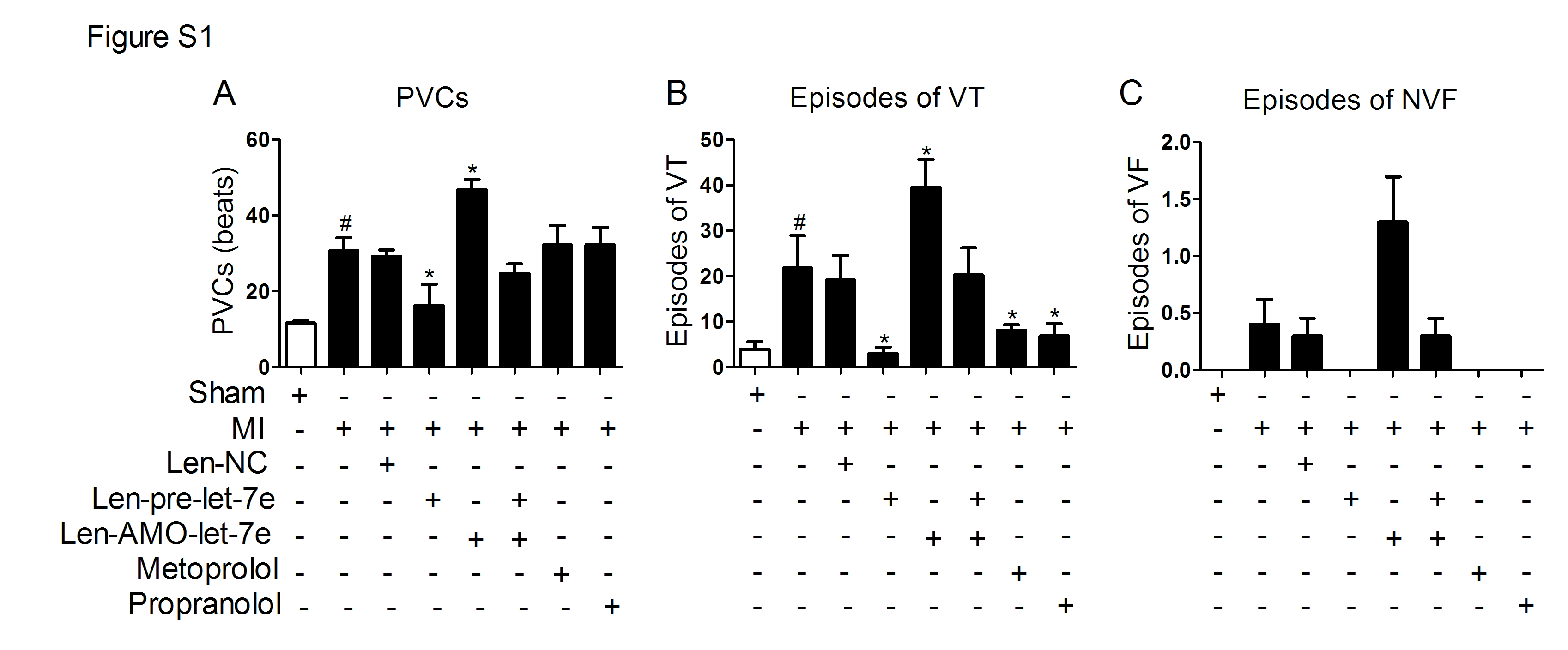

Supplement: Supplementary file 1 — Table S1 Primers used in qRT-PCR experiments. [file jcmm0018-1334-SD1.tif]
