# Supplementary material for: let-7e replacement yields potent anti-arrhythmic efficacy via targeting beta 1-adrenergic receptor in rat heart
Source: J Cell Mol Med. 2014 Apr 24;18(7):1334–43. doi: 10.1111/jcmm.12288 (PMC4124018; doi:10.1111/jcmm.12288)
Supplement: Supplementary file 2 — Figure S1 Alleviative effect of let-7e on the occurrence of PVCs, VT and NVF induced by acute myocardial infarction. [file jcmm0018-1334-SD2.doc]

**Supporting information**

Additional Supporting Information of this article:

**Table S1** **Primers used in qRT-PCR experiments.**

Gene name RT primer Forward primer Reward primer

let-7a 5’-GTCGTATCCAGTGC 5’-GGGTGAGGTAGTAG 5’-TGTCGTGGAGTCGG

GTGTCGTGGAGTCGGC GTTGTATTG-3’ CAATTG-3’

AATTGCACTGGATACG

ACAACTATAC-3’

let-7c 5’-GTCGTATCCAGTGC 5’-GGTGAGGTAGTAGG 5’-TGTCGTGGAGTCGG

GTGTCGTGGAGTCGGC TTGTATGG-3’ CAATTG-3’

AATTGCACTGGATACG

ACAACCATAC-3’

let-7d 5’-GTCGTATCCAGTGC 5’-GGGAGAGGTAGTAG 5’-TGTCGTGGAGTCGG

GTGTCGTGGAGTCGGC GTTGCA-3’ CAATTG-3’

AATTGCACTGGATACG

ACAACTATGC-3’

let-7e 5’-GTCGTATCCAGTGC 5’-GGTGAGGTAGGAGG 5’-TGTCGTGGAGTCGG

GTGTCGTGGAGTCGGC TTGTATAG-3’ CAATTG-3’

AATTGCACTGGATACG

ACAACTATAC-3’

let-7i 5’-GTCGTATCCAGTGC 5’-GGTGAGGTAGTAGT 5’-TGTCGTGGAGTCGG

GTGTCGTGGAGTCGGC TTGTGCTG-3’ CAATTG-3’

AATTGCACTGGATACG

ACAACAGCAC-3’

miR-1 5’-GTCGTATCCAGTGC 5’-GGGGTGGAATGTAA 5’-CGTGGAGTCGGCAA

GTGTCGTGGAGTCGGC AGAAGTG-3’ TTGCA-3’

AATTGCACTGGATACG

ACATACACA-3’

U6 5’-CGCTTCACGAATTTG 5’-GCTTCGGCAGCACA 5’-CGCTTCACGAATTTG

CGTGTCAT-3’ TATACTAAAAT-3’ CGTGTCAT-3’

β_1_-AR Random primer 5’-AGCGCCGATCTGGT 5’-GACACACAGGGTCT

CATG-3’ CGATGCT-3’

GAPDH Random primer 5’-AAGAAGGTGGTGAA 5’-TCCACCACCCAGTT

GCAGGC-3’ GCTGTA-3’

**Figure S1 Alleviative effect of let-7e on the occurrence of PVCs, VT and NVF induced by acute myocardial infarction.** Episodes of premature ventricular arrhythmias (PVCs) (A), ventricular tachycardia (VT) (B) and ventricular fibrillation terminated not spontaneous (NVF) (C) in different groups. Date are expressed as mean ± SEM, n=10 rats in each group; ^#^*p*<0.05 *vs* Sham, ^*^*p*<0.05 *vs* MI.
